# Development and validation of a survival model for lung adenocarcinoma based on autophagy-associated genes

**DOI:** 10.1186/s12967-020-02321-z

**Published:** 2020-04-01

**Authors:** Xiaofei Wang, Shuang Yao, Zengtuan Xiao, Jialin Gong, Zuo Liu, Baoai Han, Zhenfa Zhang

**Affiliations:** Department of Lung Cancer Surgery, Tianjin Medical University Cancer Institute and Hospital, Huanhu West Rd, Tianjin, China

**Keywords:** LUAD, Autophagy-associated genes, Survival, The Cancer Genome Atlas

## Abstract

**Background:**

Given that abnormal autophagy is involved in the pathogenesis of cancers, we sought to explore the potential value of autophagy-associated genes in lung adenocarcinoma (LUAD).

**Methods:**

RNA sequencing and clinical data on tumour and normal samples were acquired from The Cancer Genome Atlas (TCGA) database and randomly assigned to training and testing groups. Differentially expressed autophagy-associated genes (AAGs) were screened. Within the training group, Cox regression and Lasso regression analyses were conducted to screen five prognostic AAGs, which were used to develop a model. Kaplan–Meier (KM) and receiver operating characteristic (ROC) curves were plotted to determine the performance of the model in both groups. Immunohistochemistry was used to demonstrate the differential expression of AAGs in tumour and normal tissues at the protein level. Gene Ontology (GO) functional annotation and Kyoto Encyclopedia of Genes and Genomes (KEGG) pathway enrichment analyses were utilized to further elucidate the roles of AAGs in LUAD.

**Results:**

The data from the TCGA database included 497 tumour and 54 normal samples, within which 30 differentially expressed AAGs were screened. Using Cox regression and Lasso regression analyses for the training group, 5 prognostic AAGs were identified and the prognostic model was constructed. Patients with low risk had better overall survival (OS) in the training group (3-year OS, 73.0% vs 48.0%; 5-year OS, 45.0% vs 33.8%; P = 1.305E−04) and in the testing group (3-year OS, 66.8% vs 41.2%; 5-year OS, 31.7% vs 25.8%; P = 1.027E−03). The areas under the ROC curves (AUC) were significant for both the training and testing groups (3-year AUC, 0.810 vs 0.894; 5-year AUC, 0.792 vs 0.749).

**Conclusions:**

We developed a survival model for LUAD and validated the performance of the model, which may provide superior outcomes for the patients.

## Background

Lung cancer has the highest morbidity and mortality rates worldwide and is therefore a constant threat to human life [1]. Lung adenocarcinoma (LUAD) is a prevalent pathological subtype of lung cancer, accounting for nearly 45% of lung cancer. Despite advances in the global medical industry and changes in health awareness, the outcomes of patients with lung cancer remain poor, in part because almost 80% of the patients are at an advanced stage when diagnosed; another reason may be that the current TNM (tumour size/lymph nodes/distant metastasis) staging system is not always accurate for postoperative tumour staging, and therefore necessary adjuvant treatments may not be applied [2, 3]. Therefore, it is necessary to explore alternative methods for diagnosis and accurate postoperative tumour staging.

Autophagy is considered a vital catabolic process within eukaryotic cells, allowing lysosomes to degrade damaged, senescent, or nonfunctional proteins and organelles [4, 5]. Early studies have reported that autophagy is involved in many pathophysiological processes such as immune responses, inflammation, neurodegenerative diseases, tumourigenesis and cancer progression [6, 7]. Early in 1976, JS et al. first reported that cellular autophagocytosis progressed in cervical cancer cells in the absence of serum and amino acids [8]. Later studies showed that autophagy may play a part in degrading and recycling components of nonfunctional organelles to supply the demands of tumour progression [9, 10]. Nassour et al. demonstrated that autophagy was vital for tumour suppression, and the absence of autophagy was necessary for the initiation of tumours [11]. Therefore, autophagy may not only be involved in the inhibition of cancer but may also be related to the development and advancement of tumours [12–14].

Over the last decade, scholars have performed many studies to explore the role of autophagy in LUAD. Some studies have concluded that downregulating autophagy indirectly enhances the efficacy of the LUAD suppressors [15–17]; conversely, high-level autophagy was proven to promote tumourigenesis of LUAD in other studies [18–22]. Some results have provided evidence that the upregulation of autophagy is correlated with cisplatin or docetaxel resistance in LUAD [23–25]. Wang et al. found that autophagy impacted the low-dose hyper-radiosensitivity of LUAD [26].

Given these contradictory results, we sought to explore the potential value of autophagy in LUAD by integrating the entire set of autophagy-associated genes (AAGs) and the corresponding gene expression with clinical data acquired from The Cancer Genome Atlas (TCGA) portal. First, 30 AAGs that were differentially expressed in tumour and non-tumour tissues were screened and randomly divided into training and testing groups. We then performed Cox regression and Lasso regression analyses within the training group to identify the AAGs associated with remarkable overall survival (OS) in LUAD patients, and the prognostic model was constructed. To validate the accuracy of the model, the Kaplan–Meier (KM) estimator and the receiver operating characteristic (ROC) curve were applied. In addition, we investigated the results of Gene Ontology (GO) functional annotation and Kyoto Encyclopedia of Genes and Genomes (KEGG) pathway analyses to further elucidate the role of AAGs in LUAD.

## Materials and methods

### Data source and pre-processing

The entire set of 232 AAGs was collected from the human autophagy portal (http://www.autophagy.lu/index.html), which is an online database that provides a complete set of human genes related to autophagy as described in the literature. RNA sequencing and clinical data consisting of 497 LUAD and 54 non-tumour tissues was downloaded from the TCGA data portal. The ensemble gene IDs were then converted to gene symbols using the online database GENCODE (https://www.gencodegenes.org/human/releases.html), a project for referencing human genome annotation. Finally, the expression data of the AAGs were extracted.

### Screening of differentially expressed AAGs in LUAD

The expression data of 232 AAGs comprising 497 LUAD and 54 non-tumour samples were processed using the mean function, and the mean expression values were normalized by log2 transformation. The 30 AAGs that were differentially expressed between the tumour and normal samples were then identified using the Wilcoxon signed-rank test in R (version 3.6.1, https://www.r-project.org/) with a threshold of |log(fold change) > 1 and a false discovery rate (FDR) < 0.05. Next, we integrated the expression data of the 30 AAGs with the corresponding clinical information. Finally, the data were randomly divided into training and testing groups for subsequent validation.

The expression data of the 30 AAGs within the training group were then analysed using univariate Cox regression analysis to obtain the AAGs that were significantly related to survival (P < 0.05). The least absolute shrinkage and selection operator (Lasso) regression selectively enters variables into the model to obtain improved performance parameters and to control the complexity of the model through a series of parameters to avoid overfitting [27]. Therefore, we employed a Lasso regression analysis to remove highly correlated survival-related AAGs.

### Construction of the prognostic model

We performed a multivariate Cox regression analysis using both forward and backward selection to identify the 5 prognostic AAGs and their coefficients, on which we constructed the prognostic model. Every LUAD patient in both training and testing groups received an individual risk score.

The calculation of the risk score based on the AAG model was conducted as follows: $Risk \, score \, = \mathop \sum \nolimits_{{\varvec{i} = 1}}^{\varvec{n}} \varvec{v}_{\varvec{i}} \times \varvec{c}_{\varvec{i}}$ (the $\varvec{v}_{\varvec{i}}$ is the expression value of gene ***i***, $\varvec{c}_{\varvec{i}} \varvec{ }$ represents the regression coefficient of gene ***i*** in the multivariate Cox regression analysis, and ***n*** represents the number of independent indicators).

### Validating the performance of the prognostic model in training and testing groups

Based on the individual risk scores, all patients were separated into one of two groups (low/high score) by the median risk scores. The Kaplan–Meier (K-M) survival curve was plotted to evaluate the differences in overall survival between the two groups using the log-rank test to assign statistical significance. In addition, we generated receiver operating characteristic (ROC) curves to determine the accuracy of the prognostic model.

### Exploration of the expression of AAGs at the protein level

The Human Protein Atlas is an interactive web-based database (https://www.proteinatlas.org) that contains the RNA and protein expression profiles of more than ninety percent of the putative protein-encoding genes and includes more than 13 million high-resolution images [28]. The immunohistochemical results of the five prognostic AAGs were explored using this database to verify their differential expression in tumour and normal tissues.

### Enrichment analysis of AAGs

To explore the potential tumour-related molecular mechanisms of AAGs, GO functional annotation and KEGG pathway enrichment analyses were performed in R using the packages DOSE, Cluster Profiler, ggplot2, GO plot, etc. with both the p-value and q-value set at 0.05. The outcomes were visually illustrated in multidimensional views.

### Statistical analysis

All statistical analyses and graphics were performed using the R 3.6.1 (https://www.r-project.org/) and Perl language packages. Cox regression analyses were utilized to screen the AAGs related to survival. A Lasso regression analysis was used to eliminate highly correlated AAGs and prevent overfitting of the model. The Kaplan–Meier curve was plotted to display the differences in overall survival between the two groups and the log-rank test was performed to determine the significance of the differences. The ROC curve and the corresponding area under the curve (AUC) were used to evaluate the performance of the model. Statistical significance was defined as P < 0.05.

## Results

### Differentially expressed AAGs in lung adenocarcinoma (LUAD)

**Fig. 1:**
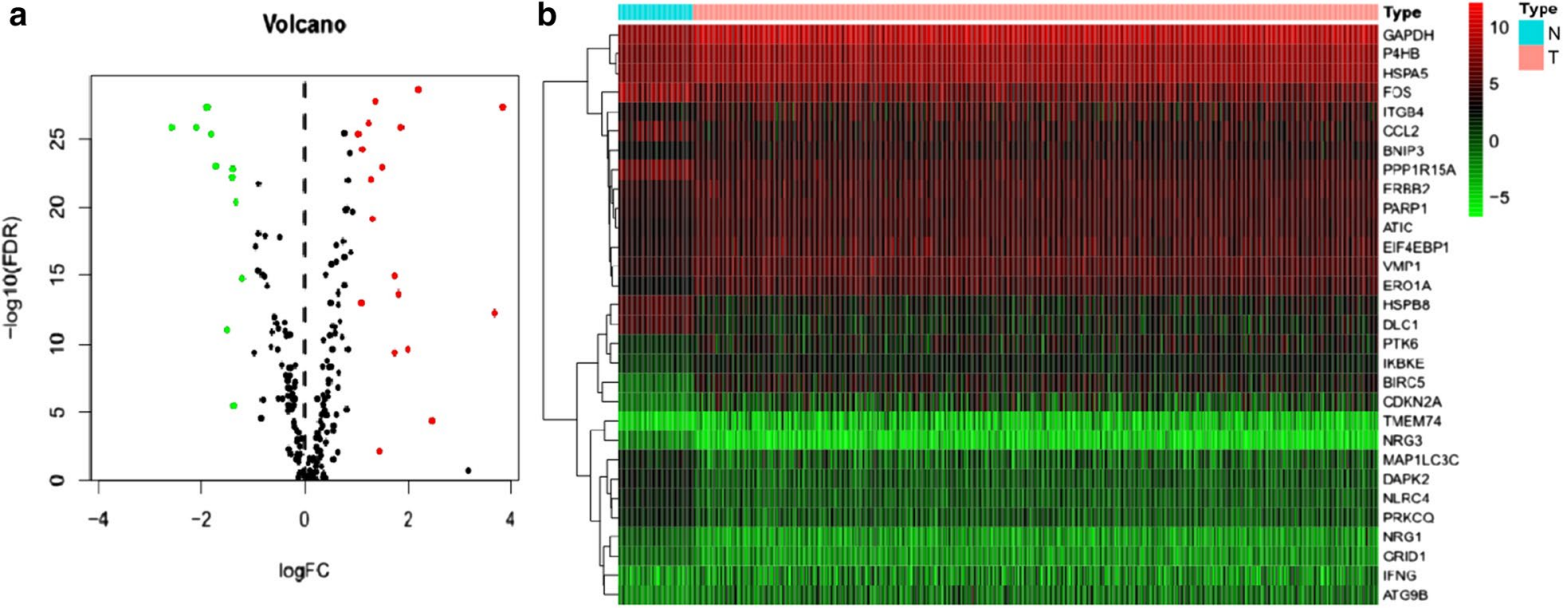
Differentially expressed autophagy-associated genes (AAGs) in lung adenocarcinoma (LUAD) and non-tumour samples. **a** The volcano map of 232 AAGs. The red dots indicate genes with high expression and the green dots represent genes with low expression. **b** Hierarchical clustering distribution of differentially expressed AAGs in normal and tumour samples

**Fig. 2:**
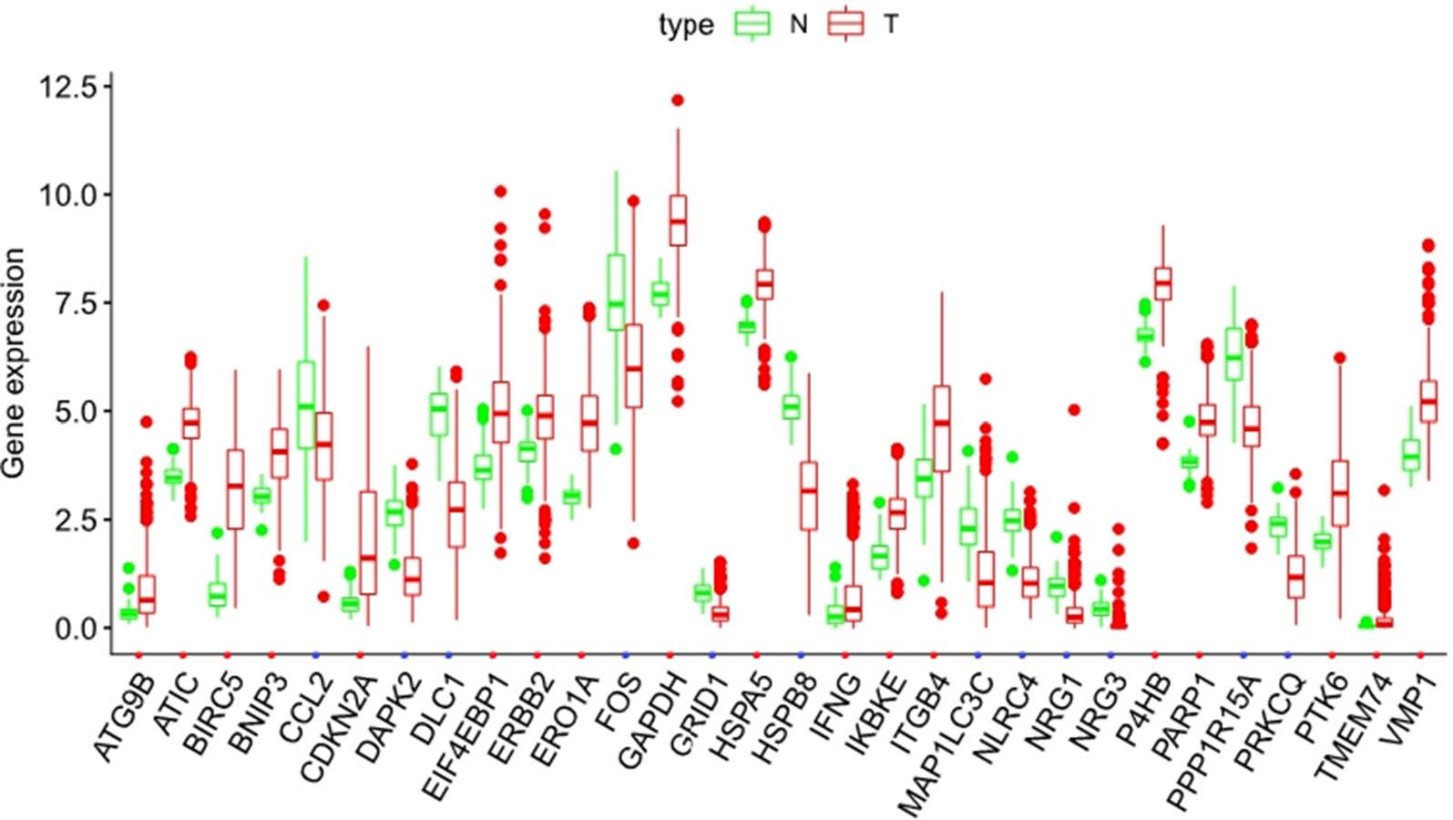
Boxplots of the expression levels of 30 autophagy-associated genes (AAGs) in tumour and normal tissues. The red box plots above the corresponding gene name represent the expression in tumour samples, whereas the green box plots represent the expression in normal samples; the red dots on the X-axis indicate genes with high levels of expression and the blue dots indicate genes with low levels of expression. (Difference analysis by Wilcoxon signed-rank test and all false discovery rate (FDR) < 0.05)

We analysed the expression of 232 AAGs in 497 LUAD and 54 non-tumour tissues using the Wilcoxon signed-rank test in R, and 30 AAGs were eventually identified using the criteria of |log2FC| > 1 and FDR < 0.05, including 12 downregulated genes (NRG3, DLC1, NLRC4, HSPB8, DAPK2, PPP1R15A, FOS, NRG1, PRKCQ, CCL2, GRID1, MAP1LC3C) and 18 upregulated genes (HSPA5, ERBB2, PARP1, P4HB, IKBKE, BNIP3, ATIC, IFNG, VMP1, ITGB4, EIF4EBP1, PTK6, GAPDH, ATG9B, ERO1A, TMEM74, CDKN2A, BIRC5) (Fig. 1). The ggpubr package in R was utilized to exhibit the expression patterns of the 30 AAGs in tumour and normal samples. The red box plots above the gene names represent tumour samples and the green box plots represent normal samples (Fig. 2).

### Survival-related AAGs and the prognostic model

**Fig. 3:**
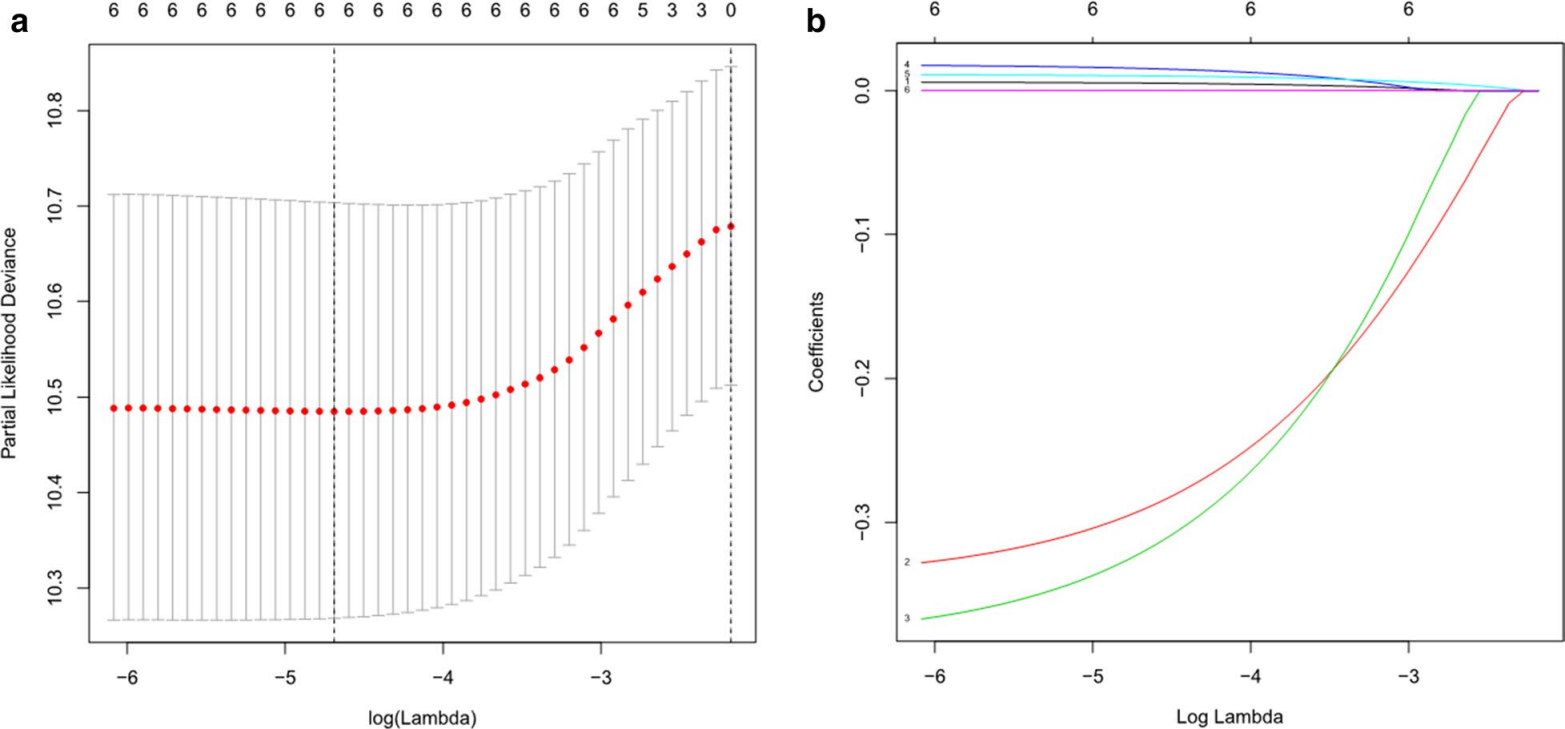
Screening of the optimal AAGs used for the final construction of the predictive model using a Lasso regression. **a** Screening of optimal parameter (lambda) at which the vertical lines were drawn. **b** Lasso coefficient profiles of the six AAGs with non-zero coefficients determined by the optimal lambda

We conducted a univariate Cox regression analysis and identified 6 AAGs (GAPDH, ERO1A, NLRC4, ITGB4, ATG9B, and CDKN2A) that were significantly related to LUAD survival in the training group. Of the 6 survival-related AAGs, 4 genes (GAPDH, ERO1A, ITGB4, and CDKN2A) were considered risk factors (all P < 0.05; HRs, 1.0007–1.0175) and that their overexpression may reduce survival; overexpression of the remaining two genes (NLRC4 and ATG9B) (all P < 0.05; HRs, 0.6913 and 0.7382, respectively) may improve the survival of patients. The Lasso regression analysis was then applied to exclude genes that may be highly correlated with other genes (Fig. 3). The 6 survival-related AAGs were then submitted to a multivariate Cox proportional hazards model, resulting in 5 candidate genes (ITGB4, NLRC4, ATG9B, CDKN2A, and ERO1A) that may serve as significant predictors of the prognosis (Table 1). Based on the 5 candidate AAGs, the formula for the risk score of every LUAD patient was constructed: *risk score* = (expression value of ITGB4 * 0.0063) + (-expression value of NLRC4 * 0.354) + (-expression value of ATG9B * 0.3956) + (expression value of CDKN2A * 0.0202) + (expression value of ERO1A * 0.0122).

**Table 1:** Univariate and multivariate cox regression analyses of OS in lung adenocarcinoma patients

Genes	Univariate analysis		Multivariate analysis		
	HR (95% CI)	P	HR (95% CI)	P	Coef
ITGB4	1.0061 (1.0005–1.0117)	0.0328	1.0064 (1.0005–1.0123)	0.0338	0.0063
NLRC4	0.6913 (0.5023–0.9513)	0.0234	0.7019 (0.5020–0.9813)	0.0384	− 0.354
ATG9B	0.7382 (0.5583–0.9761)	0.0332	0.6733 (0.5007–0.9054)	0.0088	− 0.3955
CDKN2A	1.0175 (1.0000–1.0353)	0.0495	1.0204 (1.0025–1.3868)	0.0252	0.0202
ERO1A	1.0133 (1.0056–1.0212)	0.0008	1.0123 (1.0040–1.0206)	0.0035	0.0122
GAPDH	1.0007 (1.0003–1.0010)	0.0004			

*HR* hazard ratio, *OS* overall survival, *Coef* regression coefficient of genes in the multivariate Cox regression analysis

### Validation of the model performance

**Fig. 4:**
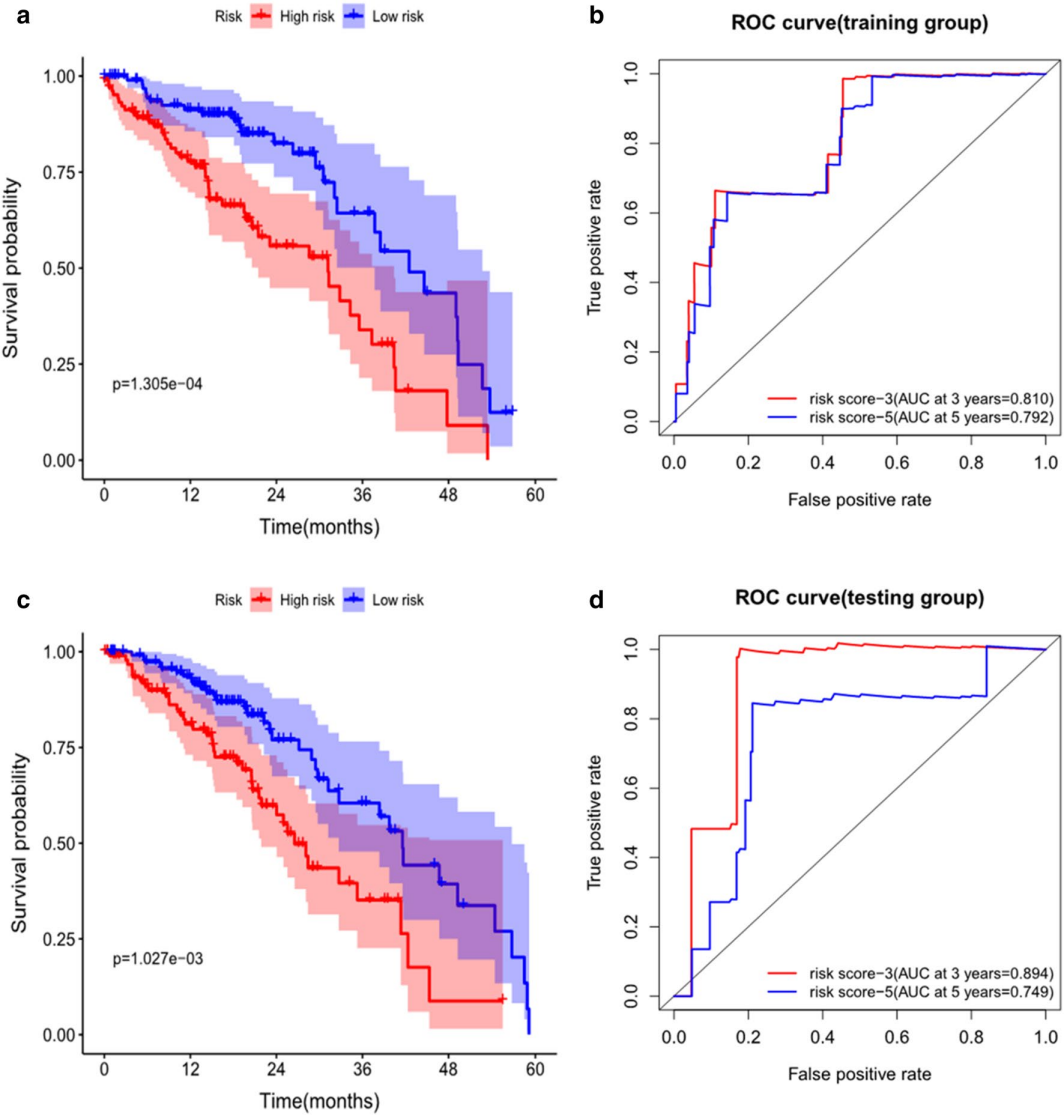
**a** K-M curve of the high-risk (red) and low-risk (blue) LUAD patients in the training group. **b** The 3-year (red) and 5-year (blue) ROC curves in the training group of LUAD patients. **c** K–M curve of the high-risk (red) and low-risk (blue) LUAD patients in the testing group. **d** The 3-year (red) and 5-year (blue) ROC curves in the testing group of LUAD patients

To validate the accuracy of the model, we plotted the KM survival curve to evaluate the difference in LUAD survival in both the training and testing groups. In the training group, the median overall survival of low-risk patients was 4.11 years, whereas the survival of high-risk patients was 2.86 years. In comparison, in the testing group, the median overall survival of patients with low-risk scores was 3.9 years, and the survival of patients with high-risk scores was 2.34 years. Low-risk patients exhibited higher survival than high-risk patients in the training group (3-year OS, 73.0% vs 48.0%; 5-year OS, 45.0% vs 33.8%; P = 1.305E−04) and in the testing group (3-year OS, 66.8% vs 41.2%; 5-year OS, 31.7% vs 25.8%; P = 1.027E−03) (Fig. 4a, b). Furthermore, we constructed the ROC curve to assess the accuracy of the model, and the areas under the ROC curves in the training and testing groups were both significant (3-year AUC, 0.810 vs 0.894; 5-year AUC, 0.792 vs 0.749) (Fig. 4c, d).

**Fig. 5:**
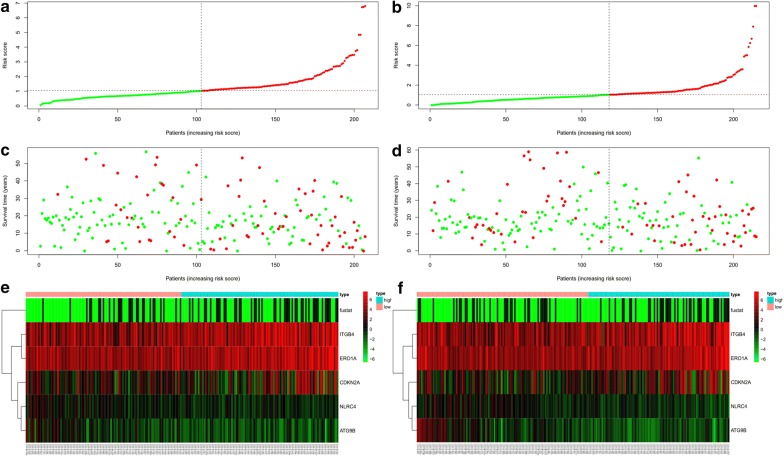
**a** Risk score distribution of LUAD patients with different risks (low, green; high, red) in the *training group*. **b** Risk score distribution of LUAD patients with different risks (low, green; high, red) in the *testing group*. **c** Scatterplots of LUAD patients with different survival status in *training group*. **d** Scatterplots of LUAD patients with different survival status in *testing group*. **e** Expression of risk genes in LUAD patients with different risks (low, pink; high, blue) in the *training group*. **f** Expression of risk genes in LUAD patients with different risks (low, pink; high, blue) in the *testing group*

In addition, we ranked the all of the LUAD patients by their risk scores to analyse the survival distribution. From the scatterplot, we could identify the survival status of patients with different risk scores; the mortality rate of patients rose with the increase in risk score. The heat maps illustrate the expression of AAGs with the rising risk scores of patients (Fig. 5a–f).

### Differential expression of prognostic AAGs at the protein level

**Fig. 6:**
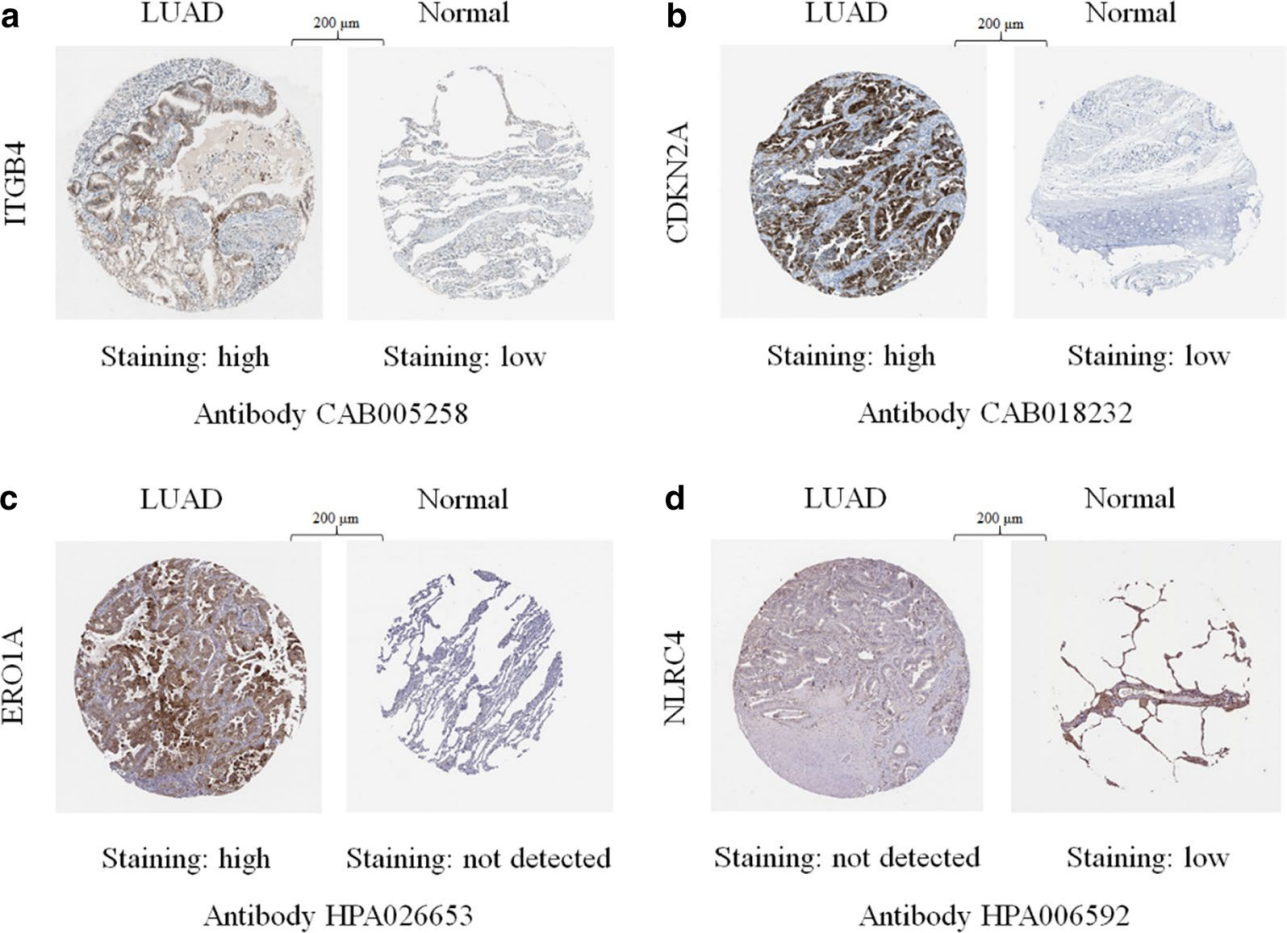
Immunohistochemistry (IHC) results showing protein levels of autophagy-associated genes in LUAD and normal tissues, **a** IHC results of ITGB4 in LUAD (staining: high; intensity: strong; quantity: 75–25%; location: cytoplasmic/membranous) and in normal tissue (staining: low; intensity: weak; quantity: 75–25%; location: cytoplasmic/membranous). **b** IHC results of CDKN2A in LUAD (staining: high; intensity: strong; quantity: > 75%; location: cytoplasmic/membranous/nuclear) and in normal tissue (staining: low; intensity: moderate; quantity: < 25%; location: cytoplasmic/membranous). **c** IHC results of ERO1A in LUAD (staining: high; intensity: strong; quantity: 75–25%; location: cytoplasmic/membranous) and in normal tissue (staining: not detected; intensity: negative; quantity: none; location: N/A). **d** IHC results of NLRC4 in LUAD (staining: not detected; intensity: negative; quantity: none; location: N/A) and in normal tissue (staining: low; intensity: moderate; quantity: < 25%; location: cytoplasmic/membranous)

We used immunohistochemistry to compare the expression of the 5 prognostic AAGs (ITGB4, NLRC4, ATG9B, CDKN2A, and ERO1A) in LUAD with their expression in normal tissues (Fig. 6a–d). As expected, the levels of protein expression of the three high-risk genes (ITGB4, CDKN2A, and ERO1A) were demonstrably higher in tumour tissues with more intense antibody staining and a greater proportion of stained cells. In contrast, NLRC4, a protective gene, stained fewer cells with weaker intensity in LUAD. The results were compatible with our findings of AAGs in LUAD; there were no data for another protective gene, ATG9B, in the Human Protein Atlas database.

### GO and KEGG analyses of AAGs

**Fig. 7:**
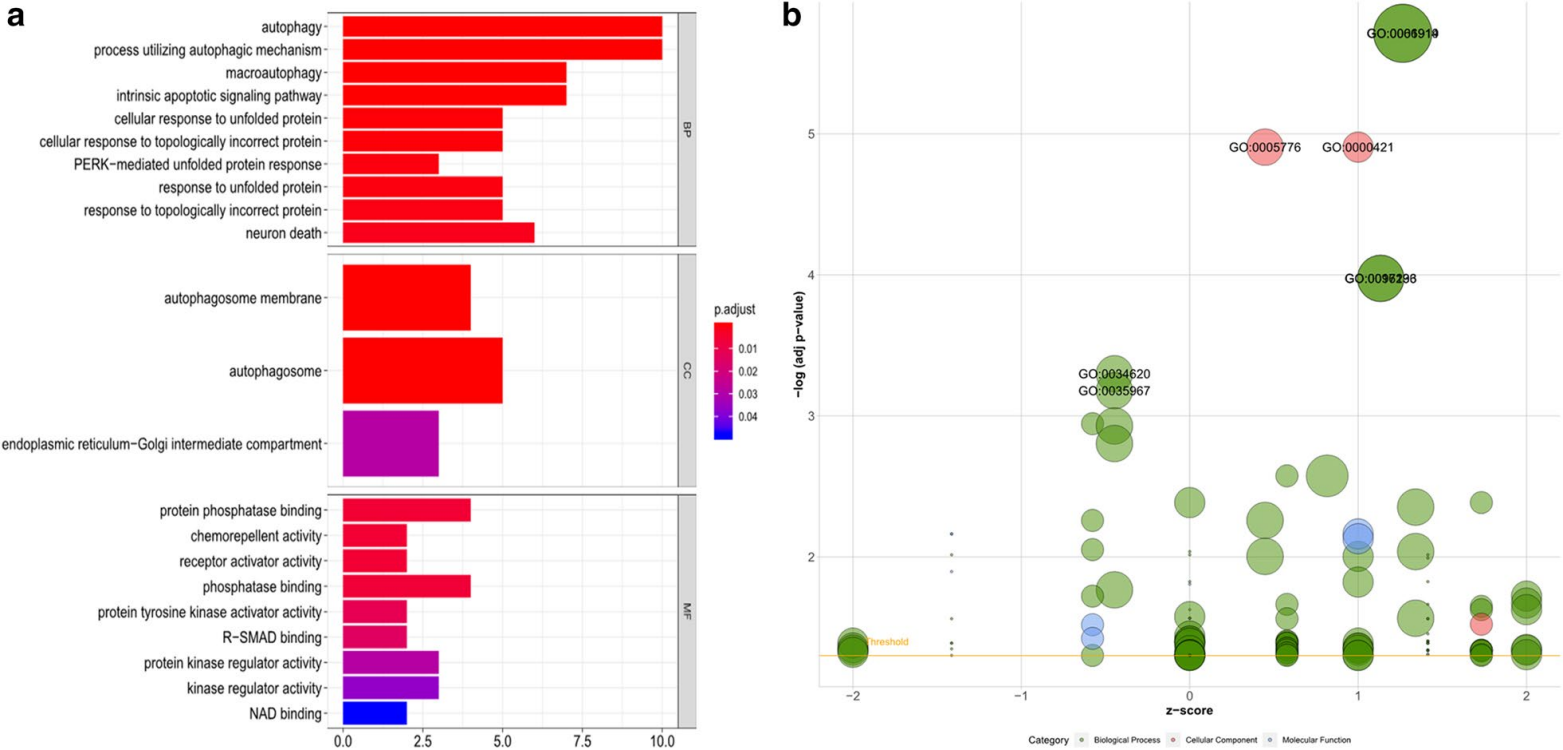
Results of Gene Ontology (GO) functional annotation analysis. **a** Bar chart of significant terms. The change in colour from blue to red represents the increase in the adjusted P-value, and the length of the bar indicates the number of gene enrichment terms. **b** Bubble plot of enriched GO terms. The Z-score is plotted on the x-axis and the -log(adjusted p-value) is plotted on the y-axis; green represents a biological process, red represents cellular components and blue represents molecular function. The size of the circles is proportional to the number of genes enriched in the term

**Fig. 8:**
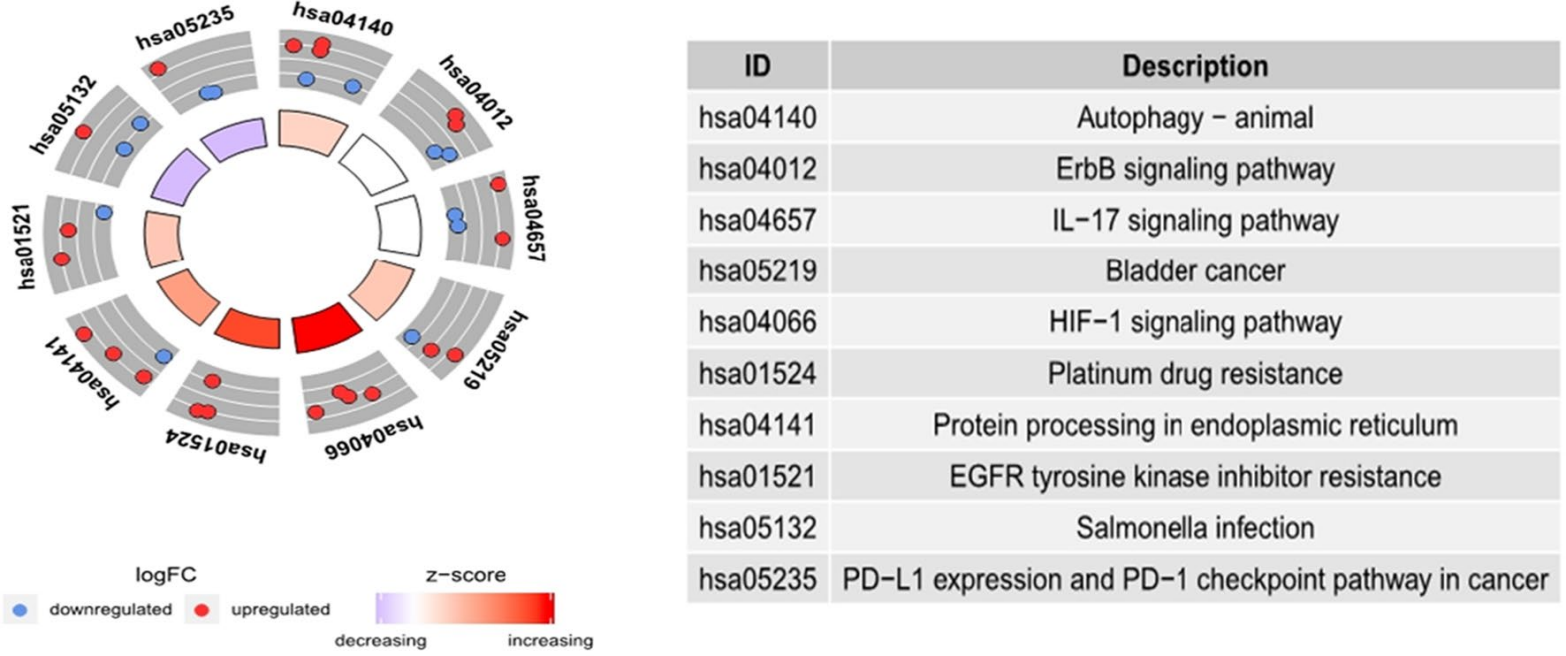
Results of Kyoto Encyclopedia of Genes and Genomes (KEGG) pathways enrichment analyses of autophagy-associated genes

To evaluate the molecular mechanisms of AAGs in LUAD, GO functional annotation and KEGG pathway enrichment analyses were conducted (Table 2). GO analysis consists of three categories: biological processes (BP), cellular components (CC) and molecular function (MF). We found that the most significant GO enriched terms involved in autophagy were the intrinsic apoptotic signalling pathway, cellular response to unfolded/topologically incorrect proteins and neuron death (BP); autophagosome and endoplasmic reticulum-Golgi intermediate compartment (CC); and protein phosphatase binding, chemorepellent activity, receptor activator activity, R-SMAD binding and NAD binding (MF) (Fig. 7a, b). In the KEGG enrichment analysis, the AAGs were primarily correlated with pathways related to autophagy-animal, the ErbB signalling pathway, bladder cancer, the HIF-1 signalling pathway, platinum drug resistance, proteins processed in the endoplasmic reticulum, EGFR tyrosine kinase inhibitor resistance, PD-L1 expression and the PD-1 checkpoint pathway in cancer (Fig. 8a, b).

**Table 2:** GO and KEGG pathway enrichment analysis of AAGs in lung adenocarcinoma

Terms	Pathway ID	Pathway description	GeneID	Count	FDR
BP	GO:0050873	Brown fat cell differentiation	BNIP3/ERO1A	2	0.04082
	GO:0010660	Regulation of muscle cell apoptotic process	BNIP3/IFNG/CDKN2A	3	0.02240
	GO:0097193	Intrinsic apoptotic signaling pathway	DAPK2/PPP1R15A/PARP1/P4HB/IKBKE/BNIP3/ERO1A	7	0.00011
	GO:0001953	Negative regulation of cell–matrix adhesion	DLC1/CDKN2A	2	0.04082
	GO:0052548	Regulation of endopeptidase activity	DLC1/NLRC4/GAPDH/BIRC5	4	0.04616
	GO:0052547	Regulation of peptidase activity	DLC1/NLRC4/GAPDH/BIRC5	4	0.04990
	GO:0034599	Cellular response to oxidative stress	FOS/PARP1/P4HB/BNIP3/ERO1A	5	0.00916
	GO:0061919	Process utilizing autophagic mechanism	HSPB8/DAPK2/MAP1LC3C/BNIP3/IFNG/VMP1/ITGB4/GAPDH/ATG9B/TMEM74	10	0.00000
	GO:0016236	Macroautophagy	HSPB8/MAP1LC3C/BNIP3/VMP1/GAPDH/ATG9B/TMEM74	7	0.00011
	GO:0035967	Cellular response to topologically incorrect protein	HSPB8/PPP1R15A/CCL2/HSPA5/ERO1A	5	0.00066
	GO:0006986	Response to unfolded protein	HSPB8/PPP1R15A/CCL2/HSPA5/ERO1A	5	0.00118
	GO:0001558	Regulation of cell growth	NRG3/PRKCQ/ERBB2/CDKN2A	4	0.04988
	GO:0006575	Cellular modified amino acid metabolic process	P4HB/ATIC/ERO1A	3	0.04632
	GO:0071456	Cellulaar response to hypoxia	P4HB/BNIP3/EIF4EBP1/ERO1A	4	0.01895
	GO:0019471	4-hydroxyproline metabolic process	P4HB/ERO1A	2	0.00966
	GO:0030968	Endoplasmic reticulum unfolded protein response	PPP1R15A/CCL2/HSPA5/ERO1A	4	0.00411
	GO:0034976	Response to endoplasmic reticulum stress	PPP1R15A/CCL2/HSPA5/P4HB/ERO1A	5	0.00551
	GO:0070059	Intrinsic apoptotic signaling pathway in response to endoplasmic reticulum stress	PPP1R15A/ERO1A	2	0.04990
CC	GO:0005776	Autophagosome	DAPK2/MAP1LC3C/VMP1/ATG9B/TMEM74	5	0.00001
	GO:0000421	Autophagosome membrane	MAP1LC3C/VMP1/ATG9B/TMEM74	4	0.00001
MF	GO:0019887	Protein kinase regulator activity	NRG3/NRG1/CDKN2A	3	0.03024
KEGG Pathways	hsa04140	Autophagy-animal	VMP1/DAPK2/BNIP3/ATG9B/PRKCQ	5	0.00940
	hsa04012	ErbB signaling pathway	NRG1/ERBB2/NLRC4/EIF4EBP1	4	0.01011
	hsa04657	IL-17 signaling pathway	CCL2/IFNG/ITGB4/FOS	4	0.01011
	hsa05219	Bladder cancer	DAPK2/CDKN2A/ERBB2	3	0.01028
	hsa04066	HIF-1 signaling pathway	IFNG/ERO1A/GAPDH/EIF4EBP1	4	0.01071
	hsa05323	Rheumatoid arthritis	CCL2/ITGB4/FOS	3	0.03726
	hsa01524	Platinum drug resistance	BIRC5/CDKN2A/ERBB2	3	0.03236
	hsa04141	Protein processing in endoplasmic reticulum	ERO1A/P4HB/PPP1R15A/HSPA5	4	0.03236
	hsa01521	EGFR tyrosine kinase inhibitor resistance	NRG1/ERO1A/EIF4EBP1	3	0.03236
	hsa05132	Salmonella infection	NLRC4/IFNG/FOS	3	0.03236
	hsa05235	PD-L1 expression and PD-1 checkpoint pathway in cancer	IFNG/FOS/CDKN2A	3	0.03726
	hsa01522	Endocrine resistance	CDKN2A/FOS/ERBB2	3	0.03988
	hsa05142	Chagas disease (American trypanosomiasis)	CCL2/IFNG/FOS	3	0.04085
	hsa04660	T cell receptor signaling pathway	IFNG/FOS/NLRC4	3	0.04085
	hsa04659	Th17 cell differentiation	IFNG/PRKCQ/NLRC4	3	0.04147

*GO* gene ontology, *KEGG* Kyoto encyclopedia of genes and genomes, *AAG* autophagy-associated genes, *FDR* false discovery rate

## Discussion

Adenocarcinoma is the most prevalent histological subtype of lung cancer and has been broadly explored for distinct genetic drivers and diverse prognostic factors. However, LUAD patients still experience high mortality due to undetected pathogenesis [29–31]. Many researchers believe that the existing guidelines and definitions of lung cancer may result in different clinical decisions for preoperative and postoperative patients. Zhang et al. found that dissection of the 4th lymph nodes was related to a better prognosis, although this was not recommended by the International Association for the Study of Lung Cancer (IASLC) [32]. There are also scholars who believe that the present staging guide is insufficient for predicting individual-level overall survival because many early-stage patients may experience a later relapse [33, 34]. Moreover, Valeria et al. suggested abandoning the concept of non-small cell lung cancer because a large body of experimental evidence suggests that LUAD and lung squamous cell carcinoma appear to be distinct tumours at the molecular, pathological and clinical levels [35]. Therefore, academics have placed increasing emphasis on the use of precision medicine in lung cancer [36–38]. It is necessary to explore methods to consolidate the current staging system and to improve the prognosis for lung cancer patients. Over the last decade, breakthroughs in microarrays and genome sequencing have promoted the discovery of prognostic biomarkers, which have greatly increased the accurate classification of diseases and improved individual treatment. Many studies have demonstrated that genomic data, particularly multigene signatures, demonstrate superior performance in prognosis analysis compared with the current staging system [39–42].

To our knowledge, this is the first study to combine the entire set of AAGs with LUAD and explore as well as validate the potential value of AAGs in LUAD. First, we selected 30 differentially expressed AAGs from 497 tumour samples and 54 normal samples. We then randomly divided the data into training and testing groups. Using Lasso regression and Cox survival analyses, we constructed a risk model based on five prognostic AAGs (ITGB4, NLRC4, ATG9B, CDKN2A, and ERO1A). Using the model, every LUAD patient was assigned a risk score. The differences in survival between patients with low and high scores were significant in both the training group and the testing group. The ROC curves and AUCs indicated that models of the training and testing groups both performed well. In addition, we performed immunohistochemistry that further proved the significant roles of AAGs in LUAD. Furthermore, GO and KEGG enrichment analyses of the differentially expressed AAGs were conducted to explore the underlying molecular mechanisms. The results of GO functional annotation revealed that the AAGs were primarily enriched in the intrinsic apoptotic signalling pathway, cellular response to topologically incorrect proteins and the PERK-mediated unfolded protein response, which is consistent with the conclusion of previous studies that autophagy is a physiological process that eliminates misfolded proteins and damaged organelles in response to cellular stress [12, 43]. In the KEGG pathway analysis, AAGs were primarily enriched in the ErbB, IL-17 and HIF-1 signalling pathways. EGFR (ErbB1) is not unknown to us; in 2004, there was a major discovery that treatment with the EGFR-TKI (epidermal growth factor receptor-tyrosine kinase inhibitor) gefitinib caused tumour regression in some patients with NSCLC [44], and the third-generation EGFR-TKI axitinib confers greater survival benefits to patients, particularly those with the T790M mutation [45]. In addition to EGFR (ErbB1), the proteins HER2 (ErbB2), HER3 (ErbB3) and HER4 (ErbB4) compose the ErbB family of transmembrane receptor tyrosine kinases (RTKs), which is one of the most broadly explored therapeutic targets in human malignancies [46]. Jutten et al. found that autophagy activity influenced the expression of EGFR and the resistance to EGFR-targeting therapies could be reduced by downregulating autophagy [47, 48]; IL-17 (interleukin-17), as a signature proinflammatory cytokine of the CD4+ T helper 17 (Th17) cells [49], was shown to participate in the formation and advancement of various tumours [50] and was widely distributed in the tumour microenvironment, where it has twin roles in tumourigenesis and tumour suppression [51]. Previous studies have indicated that the formation of lung cancer is closely related to local dysbiosis and inflammation mediated by Th17 cells [52, 53]. The 2019 Nobel Prize in physiology or medicine was awarded to Professor William G. Kaelin Jr., Sir Peter J. Ratcliffe and Gregg L. Semenza for their contributions to elucidating the mechanisms by which cells sense and adapt to the availability of oxygen. They found that HIF-1 (hypoxia-inducible factor-1) regulates more than 4000 targeted genes, some of which can increase oxygen transport for angiogenesis and erythropoiesis. Another study reported that under emergent oxygen fluctuations, autophagy can be harmful and can lead to cell death [54]. Moreover, Bellot et al. reported that various mechanisms such as autophagy activation enabled tumour cells to adjust to hypoxia [55]. Therefore, the regulation of HIF-1 may represent an important breakthrough in tumour therapy, as angiogenesis and erythropoiesis play crucial roles in the occurrence and development of cancer.

It is apparent that autophagy plays many roles in tumourigenesis and development, which is consistent with the association between autophagy genes and LUAD in our study. However, some limitations are worth noting. As a retrospective study, this research has an inherent bias; although we validated the model using training/testing sets and immunohistochemistry, additional validation of prognostic designs should be conducted in vitro, in vivo and in clinical trials; moreover, the biological processes and molecular mechanisms of the 5 AAGs should be further evaluated to accelerate their clinical application in LUAD.

## Conclusions

In this study, we provided insights into the roles of autophagy genes in LUAD and constructed a promising model, which could provide a reference to determine whether postoperative/preoperative patients are at high risk. These patients are then more likely to receive more comprehensive neoadjuvant/adjuvant therapy with an improved prognosis.

## Data Availability

All data used in this study were acquired from The Cancer Genome Atlas (TCGA) portal.

## Abbreviations

LUAD: Lung adenocarcinoma
TCGA: The Cancer Genome Atlas (database)
ROC: Receiver operating characteristic
AUC: The area under the ROC curve
TNM: Tumour size/lymph nodes/distance metastasis, a tumour staging system used in oncology and constructed by the American Joint Committee on Cancer and the Union for International Cancer Control
GO: Gene Ontology
KEGG: Kyoto Encyclopedia of Genes and Genomes
FDR: False discovery rate
IASLC: International Association for the Study of Lung Cancer
